# *Saccharomyces cerevisiae* Fermentation Products That Mitigate Foodborne *Salmonella* in Cattle and Poultry

**DOI:** 10.3389/fvets.2019.00107

**Published:** 2019-04-10

**Authors:** Kristina M. Feye, Jasmine P. Carroll, Kristi L. Anderson, John H. Whittaker, Garrett R. Schmidt-McCormack, Don R. McIntyre, Hilary O. Pavlidis, Steve A. Carlson

**Affiliations:** ^1^Department of Biomedical Sciences, Iowa State University College of Medicine, Ames, IA, United States; ^2^Diamond V, Cedar Rapids, IA, United States

**Keywords:** *Salmonella*, antibiotic resistance, virulence, yeast fermentation products, food safety

## Abstract

Prior studies revealed that yeast fermentation products, specifically XPC™ and related products (Diamond V, Cedar Rapids, IA), serve as viable food safety tools across multiple food animal species including cattle and poultry. Providing this supplement in feed leads to reduced prevalence, load, virulence, and antibiotic resistance of foodborne pathogens such as *Salmonella* and *Escherichia coli* O157:H7. These findings are worthy of further study, especially when coupled with the enhanced growth and performance observed with these products. Mechanistically, XPC appears to modulate these effects through the immune system and gut microbiome. Herein we further investigated this product and demonstrate that XPC mediates an enhancement of immunocyte killing of *Salmonella* in calves fed the product. Additionally, these studies reveal that XPC reduces the lymph node infiltration, invasiveness, and antibiotic resistance of *Salmonella* in dairy calves fed the product-consistent with findings observed in poultry and adult beef cattle. Furthermore, the reduction in invasiveness does not lead to a rebound hyperinvasive phenotype in *Salmonella* obtained from XPC-fed animals. In summary, these studies suggest that XPC reduces the invasion of *Salmonella* and may alter various phenotypic characteristics of the pathogen.

## Introduction

The presence of important foodborne pathogens continues to prove a formidable challenge to overcome. Two important foodborne pathogens, *Salmonella enterica* and shiga toxin-producing *Escherichia coli* serotypes such as O157:H7 (STEC), are serious concerns in food animal production management.

Yeast fermentation products, specifically the *Saccharomyces cerevisiae* fermentation product designated as XPC™, appears to impact enteric pathogens across multiple livestock species (1–3). Traditionally, XPC has been used for its beneficial production effects (4, 5). However, studies into its anti-pathogenic effects began after anecdotal observations indicated that there was a decrease in environmental *Salmonella* in chicken laying barns as part of the National Poultry Improvement Plan environmental monitoring. Questions began to emerge as to the potential use and efficacy of XPC as a pathogen mitigation tool, as well as the mechanisms underlying its effects. Recent and ongoing studies suggest that XPC, and derivatives, is a viable intervention with uses in food animal production (1–3).

Given the anecdotal reports of XPC-mediated reduction of *Salmonella* in poultry, the anti-*Salmonella* effects of XPC were examined in dairy calves. An investigator-blinded study addressed the hypothesis that XPC plus SmartCare™, an XPC derivative that is included in milk replacer, reduces the impact of *Salmonella* in dairy calves on milk (1). This study indicated that the consequences of feeding calves XPC plus SmartCare are an overall improvement of gastrointestinal health and development as well as a significant reduction in *Salmonella* load and virulence (1).

The positive results from the dairy calf study prompted an analogous study in beef cattle using an XPC derivative specific for those animals and is designated as NaturSafe™. This investigator-blinded study examined the effects of NaturSafe on fecal shedding, lymph node colonization, virulence, and antibiotic resistance of *Salmonella* as well as the prevalence and load of STEC (2). The results indicated that NaturSafe mediated a decrease in *Salmonella* load, a decrease in *Salmonella* fecal shedding, and a decrease in *Salmonella* colonization of the lymph nodes when compared to the Control diet (2).

Another study was conducted to examine the anti-*Salmonella* properties of XPC in regards to the load, prevalence, virulence, and antibiotic resistance of *Salmonella* in a controlled experiment with broilers. These studies revealed an XPC-mediated reduction of load and prevalence of *Salmonella* as well as a reduction in virulence and antibiotic resistance (3).

The aforementioned studies prompted further investigations into the anti-*Salmonella* effects of XPC. Specifically, the studies presented herein were designed to address the following hypotheses: XPC plus SmartCare enhances the immune function of calves whereby *Salmonella* is cleared; the feeding of XPC plus SmartCare leads to anti-virulence and anti-antibiotic resistance effects on *Salmonella* present in calves; and, the XPC-mediated inhibition of virulence is sustained and does not lead to an exaggerated rebound effect once the pathogen exits an XPC-fed animal.

## Materials and Methods

### Assessment of Immune Clearance of *Salmonella* in Dairy Calves

All animal experiments were approved by the Institutional Animal Care and Use Committee at Iowa State University. Newborn dairy calves (*n* = 20 newborn Holstein heifers and bulls in each treatment group) were fed daily doses of XPC plus SmartCare for 2 weeks as part of a previous study (1). Prior to experimental infection with *Salmonella*, approximately 4 mL of whole blood was collected into an EDTA tube, of which 3 mL was transferred into a microfuge tube and subjected to density gradient centrifugation (the other 1 mL was submitted for CBC analysis). The erythrocyte fraction was then removed and 120 μL of the buffy coat interface was collected and aliquoted into six separate tubes containing RPMI media, to which 10^7^ colony-forming units of statically grown *Salmonella* (approximate multiplicity of infection = 100; grown in LB broth) were added and the tubes were incubated at 37°C. After 1 h, extracellular (i.e., non-invasive) bacteria were killed by the addition of 50 μg/mL gentamicin. At 0, 1, 2, 4, 8, and 12 h post-killing, leukocytes were centrifuged and the gentamicin-containing media was removed and replaced with 50 μL of phosphate-buffered saline containing 1% Triton which lyses the leukocytes. Lysates were then plated on XLD agar that was incubated overnight at 37°C. The following day, black-centered colonies were enumerated and *Salmonella* survival/cell was calculated as number of recovered colonies/number of leukocytes per 20 μL blood (derived from the CBC analyses). The strains used were gentamicin-susceptible *S*. Dublin SGI1 (6) and *S*. Typhimurium LNWI (7). Studies were performed in triplicate on two occasions for each of the 40 calves used in the study (1).

These same newborn dairy calves (1) were fed daily doses of XPC plus SmartCare for 2 weeks and then experimentally infected with multiresistant *Salmonella* DT104, followed by continued feeding of XPC plus SmartCare for another 5 weeks. At the end of the study, calves were euthanized and *Salmonella* were cultured from the superficial cervical lymph nodes. The exterior surface of the lymph nodes was rinsed in ethanol, and then the lymph nodes were placed in whirl packs and smashed with a rubber mallet. An equal volume of LB broth was then added and the contents were massaged and left to sit at room temperature for 1 h. An aliquot was then plated on XLD agar and then incubated overnight at 37°C. *Salmonella* were then enumerated on XLD agar. Studies were performed in triplicate on two occasions for each of the 40 calves used in the study (1).

### Assessment of Virulence and Antibiotic Resistance of *Salmonella* in Dairy Calves

*Salmonella* isolated from dairy calves (1) were subjected to the same virulence and antibiotic resistance assays described previously (2, 3), i.e., standard tissue culture invasion assays involving HEp-2 cells (8) and micro-broth dilution antibiogram assays using MICs and resistance breakpoint concentrations. For tissue culture invasion, *Salmonella* were added to and extracted from tissue culture cells in a manner similar to that described for the leukocyte assays (except that a higher concentration of antibiotic was used since the strain is gentamicin-resistant). Antibiogram assays focused on florfenicol resistance since this resistance is encoded on an integron present in the input strain. That is, the studies presented herein assessed the ability of XPC to reduce both the invasiveness and integron-mediated antibiotic resistance of *Salmonella* obtained from dairy calves from a prior study (1). Studies were performed in triplicate on two occasions for each of the 40 calves used in the study (1). Invasion was recorded as % invasiveness, i.e., number of bacteria recovered/number of bacteria added to cells *hila* expression was recorded as an inverse of the number of PCR cycles required to visualize an amplicon. Percent resistant to florfenicol was determined as the number of colonies that grew in the breakpoint concentration (32 ug/mL) of florfenicol divided by the number of colonies assayed. Percent containing SGI was derived as the number of colonies that were PCR(+) for the SGI1 integron divided by the number of colonies assessed.

### Assessment of *Salmonella* Invasion After the Pathogen Exits an XPC-Fed Host

*Salmonella* obtained from cattle (1, 2) and broilers (3) were subjected to serial tissue culture invasion assays. Colonies were collected *en masse* and then incubated with HEp-2 tissue culture cells as per Feye et al. (3). Invasion was then determined and *hilA* expression was then quantitated (2, 3) for successive incursions into the tissue culture cells, with the bacteria recovered from cells serving as the input strain for the next round of invasion assay. Specifically, bacteria were recovered from animals on XLD plates (i.e., zero generation) and then suspended in PBS and directly added to a fresh set of HEp-2 cells. The invasion assay was then repeated and the bacteria recovered (i.e., the first generation) were used in the subsequent invasion assay. Studies were performed in triplicate for each assay, and were performed once for the calf isolates (1), once for the beef cattle isolates (2), and once for the poultry isolates (3). Data were then pooled for all three animal-specific isolates and reported as an averaged set of data. Data are compared to the wild-type statically grown strain that was not exposed to an animal.

### Statistical Analyses

For data with multiple sampling time points, a repeated measures analysis was used with the Bonferroni *ad hoc* test for multiple comparisons (Prism Graph Pad 7.0). For data with a single measurement, an analysis of variance (ANOVA) was conducted with the Tukey's *ad hoc* test for multiple comparisons.

## Results

### Immune Clearance of *Salmonella* in Dairy Calves

**Figure 1:**
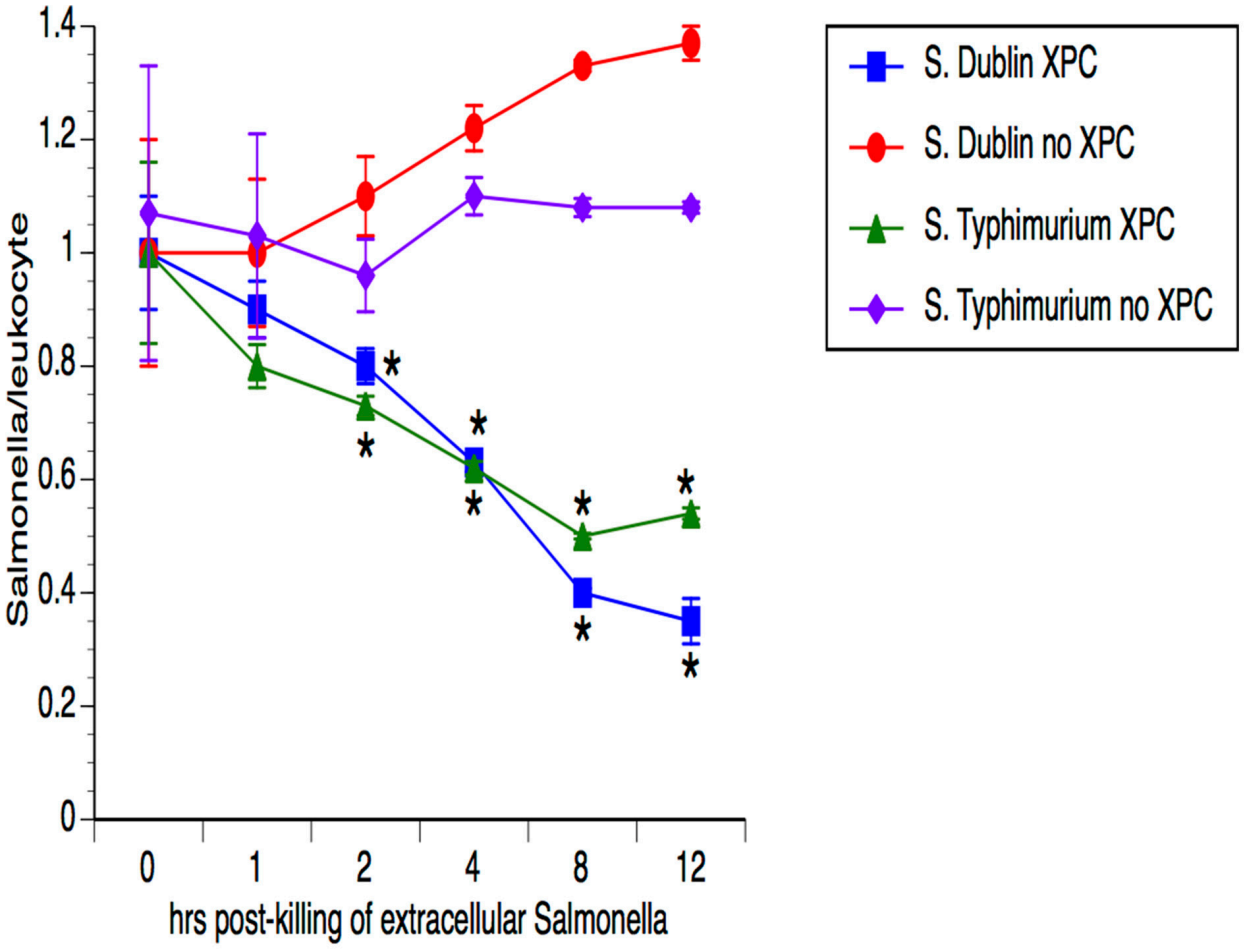
Assessment of *Salmonella* lysis by leukocytes obtained from calves fed XPC™ compared to calves that did not receive XPC. Peripheral blood cells were examined for the ability of the leukocytes to reduce the presence of *Salmonella* in calves fed XPC plus SmartCare. Specifically, whole blood was taken and incubated with *Salmonella* for 1 h. After 1 h, an enumerated portion of leukocytes were removed and exposed to gentamicin in order to kill the non-engulfed/non-invasive *Salmonella*. Leukocytes were then lysed with Triton and lysates were plated on XLD agar plates that were incubated overnight at 37°C for colony enumeration the next day. *Salmonella/leukocyte* was then calculated based on the number of *Salmonella* recovered and the number of leukocytes incubated with the *Salmonella*. This process was repeated at multiple time points in order to determine the ability of the leukocytes to temporally kill *Salmonella*. The strains used were *S*. Dublin SGI1 (6) and *S*. Typhimurium LNWI (7). Data presented are the mean ± sem from leukocytes obtained from 10 calves from each group on at least two separate occasions. *p < 0.05 vs. XPC for the same strain.

Our previous studies with SmartCare and XPC (1) revealed diminished *Salmonella* fecal shedding and intestinal carriage in calves fed the products, when compared to calves fed the Control diet. To assess the possibility that the leukocytes from XPC-fed calves are clearing the *Salmonella*, peripheral blood cells were examined for the ability of the leukocytes to temporally reduce the presence of *Salmonella*. As shown in Figure 1, the number of *Salmonella* was equivalent in both groups of calves (*n* = 5 calves/group) at the first time point but the numbers of *Salmonella* dropped significantly (50–75%) at each successive time point for leukocytes obtained from XPC-fed calves when compared to Control-fed calves. Importantly, this mechanism seems to be conserved across two divergent *Salmonella enterica* serovars- Typhimurium and Dublin.

**Figure 2:**
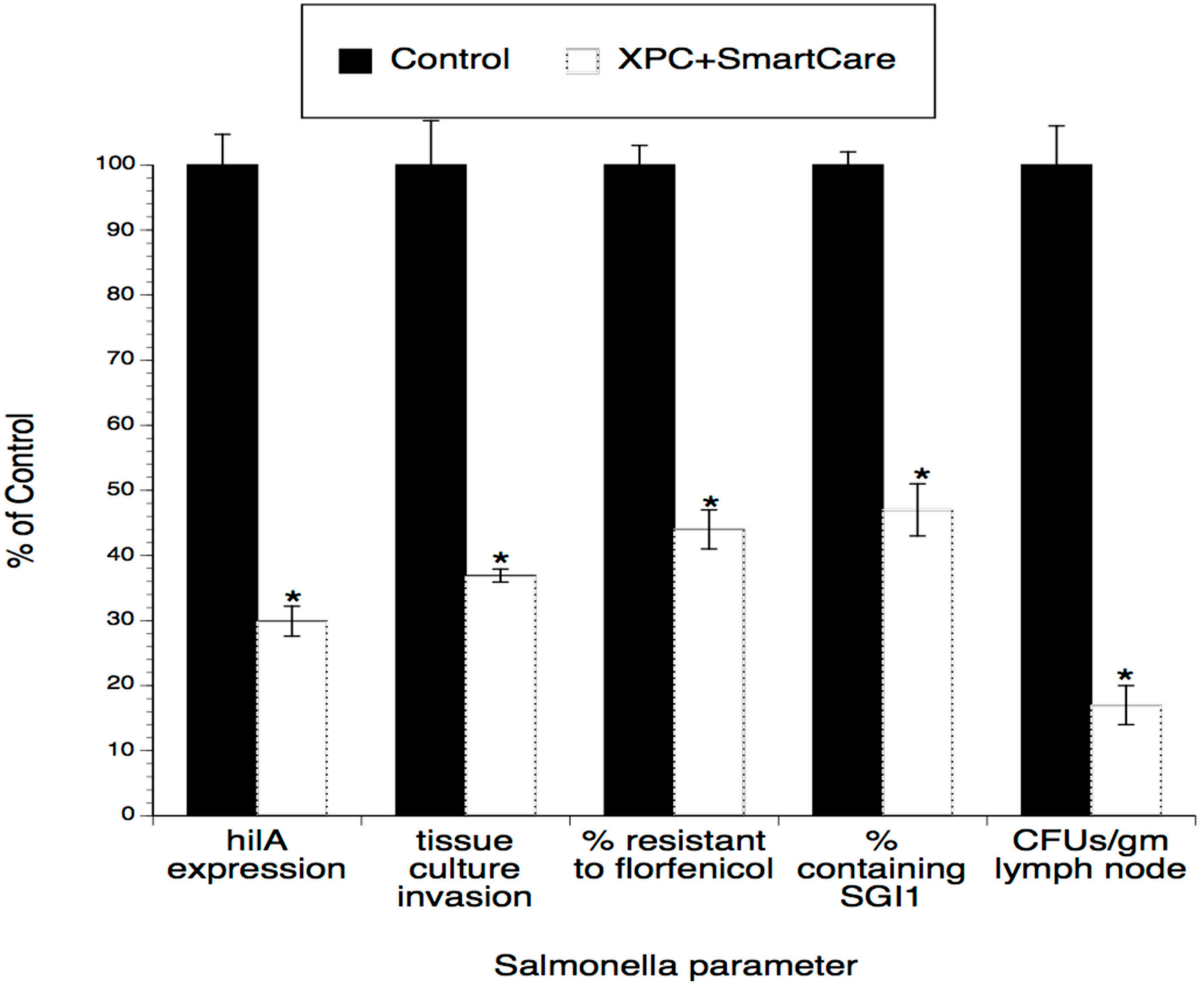
Assessment of *Salmonella* obtained from lymph nodes of calves fed XPC™ plus SmartCare™, when compared to calves that did not receive the products. Newborn dairy calves were fed daily doses of XPC plus SmartCare for two weeks and then experimentally infected with multiresistant *Salmonella* DT104, followed by continued feeding of XPC plus SmartCare for another 5 weeks (1). At the end of the study, calves were euthanized and *Salmonella* were cultured from the superficial cervical lymph nodes. *Salmonella* were enumerated and subjected to the virulence and antibiotic resistance assays described in other studies (2, 3). Data presented are the mean ± sem from data obtained from 10 calves from each group. *p < 0.05 vs. Control.

To address the possibility that these calves were more efficient at translocating the *Salmonella* to lymph nodes, peripheral lymph nodes (superficial cervical) were excised at euthanasia and subjected to selective culture for *Salmonella*. As shown in Figure 2, *Salmonella* was significantly less abundant (~80% reduced) in lymph nodes from XPC-fed calves when compared to Control-fed calves.

### Diminished Virulence and Antibiotic Resistance of *Salmonella* in Dairy Calves

**Figure 3:**
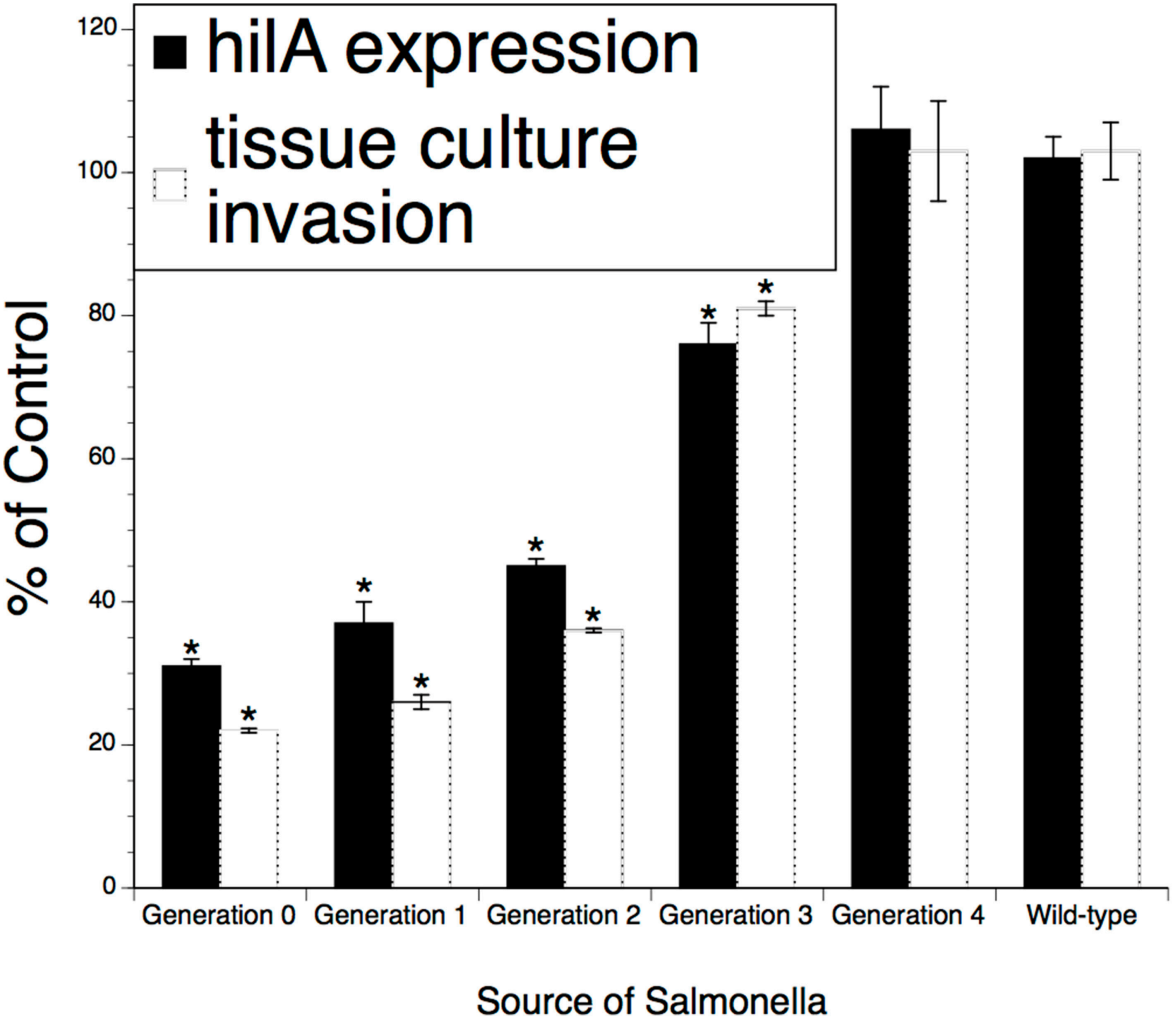
Persistence of the effect of XPC™ on the *hi/A* expression and invasiveness of *Salmonella* recovered from feces of naturally infected animals fed XPC (or a Control diet) for >28 days. *Salmonella* were recovered from treatment-specific animals (cattle and broilers) and then serially subjected to the invasion assays examining cell penetration and virulence gene expression. Generations 1–4 represent *Salmonella* serially recovered from tissue culture studies while Generation 0 represents *Salmonella* directly recovered from animals. *p < 0.05 vs. wild-type.

Since the input strain used in the poultry studies (2) was also used in the calf studies (1), similar virulence and antibiotic resistance assays were performed using *Salmonella* recovered from the calves. As shown in Figure 3, tissue culture invasiveness, *hilA* expression, antibiotic resistance, and prevalence of the antibiotic resistance element (SGI1) were all significantly diminished (~60–70% reductions) in *Salmonella* recovered from XPC-fed calves.

### Restoration of *Salmonella* Invasion After the Pathogen Exits an XPC-Fed Host

To investigate the possibility that XPC mediates a partially sustainable hypo-invasive state in *Salmonella* and the microbe will return to the fully invasive state or even become hyperinvasive after exiting an XPC-fed animal, a series of temporal invasion assays were performed on isolates recovered from XPC-fed poultry and cattle. As shown in Figure 3, invasiveness and *hila* expression were ~70–80% reduced after recovery from animals (“Generation)” and then returned to baseline after three successive incursions (designated as “Generations”) into tissue culture cells. Neither the invasiveness nor the *hila* expression went above the baseline observed for *Salmonella* recovered from Control-fed animals or from the wild-type strain. Thus, it appears that, after exiting an XPC-fed animal, the *Salmonella* need to invade three additional and subsequent hosts before returning to the fully virulent state as per the wild-type strain. These data include *Salmonella* obtained from the experimentally infected dairy calves (1), naturally infected beef cattle (2), and from poultry that were experimentally infected (3). Thus, the data represent a variety of naturally occurring *Salmonella* serotypes from a variety of hosts.

## Discussion

Herein we demonstrate that XPC mediates an enhancement of immunocyte killing of *Salmonella* in calves fed the product. Additionally, these studies reveal that XPC reduces the lymph node infiltration, invasiveness, and antibiotic resistance of *Salmonella* in dairy calves fed the product- consistent with findings observed in poultry (3) and adult beef cattle (2). Furthermore, the reduction in invasiveness does not lead to an immediate rebound hyperinvasive phenotype in *Salmonella* obtained from XPC-fed animals.

Through the implementation of XPC as a feed additive, there is an apparent advantage for reducing important foodborne illnesses attributed to food animal production. Evidence continues to show that XPC leads to a reduction in *Salmonella* prevalence, GI colonization, shedding, virulence, and antibiotic resistance across multiple species. Reducing the former three attributes will coordinately reduce the number of *Salmonella* entering the food supply. The reduced virulence in *Salmonella*, which is supported by a marked decrease in *hilA* expression and tissue culture invasiveness, has implications for both cattle and chickens where the microbe is an opportunist and a commensal, respectively. The net result is that Treated animals harbor *Salmonella* that are less efficient at causing disease in the current host or in a downstream mammalian host, thus requiring a significantly higher infectious dose for eliciting salmonellosis. XPC also directly decreases antimicrobial resistance and the benefit of this effect is 2-fold. The first benefit is a restoration of susceptibility to medically important antibiotics that occasionally need to be used for treating salmonellosis. The second benefit is a reduction of the presence of antibiotic resistance-encoding genetic elements, like plasmids and integrons, that allow for the global transfer of genetic information from commensal to pathogen and from pathogen to pathogen. Beyond transferring antibiotic resistance genotypes, these elements have been implicated in the transfer of information that improves the fitness (9) and virulence (10) of pathogenic bacteria. The mechanism for this effect is unknown, but may involve a dampening of antibiotic resistance gene expression or an elimination of the elements (plasmids or integrons).

The conserved mechanism for the anti-*Salmonella* effects of XPC is largely unknown. Research has been initiated to elucidate this interesting mechanism, though the literature is highly suggestive of two mechanisms: augmenting gut immunophysiology and enhancing microbial communities. Sustained inflammation resulting from any number of sources ultimately leads to a reduction in gut-barrier integrity, increases circulating endogenous endotoxin, and is linked to decreased feed intake and susceptibility to disease in food animals (11–13). The utilization of fermented products specifically addresses a number of these inflammatory issues by enhancing gut physiology resulting in reduced pathogenesis of important foodborne pathogens across multiple species (1, 3, 14).

XPC appears to directly enhance commensal microbial populations, leading to an improvement of bacterial competition, gastrointestinal physiology, and nutrient utilization. The underlying mechanisms governing the success of XPC may also be pertinent to the reduction of other pathogens that extend beyond the foodborne pathogens discussed herein. Therefore, XPC has the potential to be a significant and economically viable solution for curtailing foodborne pathogens and further studies will continue to enhance our understanding of postbiotics on physiology.

## Ethics Statement

This study was carried out in accordance with the recommendations of the Iowa State University Institutional Animal Care and Use Committee. The protocol was approved by the committee.

## Author Contributions

All university investigators performed experiments for the 261 study, with KF, KA and SC performing the animal experiments. The manuscript was initially authored by KF and then revised by SC. The other investigators (KA, JC, JW, GS-M, DM, and HP) then provided editorial comments that culminated in the final version of the manuscript.

### Conflict of Interest Statement

The authors declare that the research was conducted in the absence of any commercial or financial relationships that could be construed as a potential conflict of interest.
